# Probing ultrafast changes of spin and charge density profiles with resonant XUV magnetic reflectivity at the free-electron laser FERMI

**DOI:** 10.1063/1.4990650

**Published:** 2017-07-06

**Authors:** C. Gutt, T. Sant, D. Ksenzov, F. Capotondi, E. Pedersoli, L. Raimondi, I. P. Nikolov, M. Kiskinova, S. Jaiswal, G. Jakob, M. Kläui, H. Zabel, U. Pietsch

**Affiliations:** 1 Physics Department, University of Siegen, D-57072 Siegen, Germany; 2 Elettra-Sincrotrone Trieste, 34149 Basovizza, Trieste, Italy; 3 Institut für Physik, Johannes Gutenberg-Universität Mainz, D-55099 Mainz, Germany; 4 Singulus Technologies AG, 63796 Kahl am Main, Germany

## Abstract

We report the results of resonant magnetic XUV reflectivity experiments performed at the XUV free-electron laser FERMI. Circularly polarized XUV light with the photon energy tuned to the Fe M_2,3_ edge is used to measure resonant magnetic reflectivities and the corresponding *Q*-resolved asymmetry of a Permalloy/Ta/Permalloy trilayer film. The asymmetry exhibits ultrafast changes on 240 fs time scales upon pumping with ultrashort IR laser pulses. Depending on the value of the wavevector transfer *Q_z_*, we observe both decreasing and increasing values of the asymmetry parameter, which is attributed to ultrafast changes in the vertical spin and charge density profiles of the trilayer film.

## I. INTRODUCTION

The prospect of controlling magnetization on ultrafast time scales is of considerable interest since the first observation of laser induced ultrafast demagnetization by Beaurepaire *et al.*^1^ It has opened up the opportunity for light-controlled ultrafast magnetic data storage that requires profound understanding of the physics of ultrafast spin-flip processes, ultrafast spin dependent transport processes, and the complex interaction of electrons, spin, and lattice degrees of freedom in condensed matter systems. The irradiation of a magnetic sample with an ultrashort visible or IR laser pulse triggers a variety of ultrafast processes within the electronic system of the sample. A cascade of hot electrons, produced upon interactions with the photons, diffuses through the sample.^2,3^ The penetration depth of the IR radiation is limited to a few nm resulting in a strong absorption gradient in the sample and an inhomogeneous energy distribution within the same region. Since the transport properties are strongly energy and spin-dependent, this results in ultrafast spin-currents flowing across domain walls^4^ and interfaces of the magnetic layers.^5^ The hot electrons may then mediate spin-flip processes via coupling to the lattice system and they are also capable of inducing further demagnetization by transporting energy away from the IR absorption region. In this way, a spatially inhomogeneous cloud of hot spins transverses through the sample with spin-density modulations on fs time and nm length scales. To probe these and thus understand the ultrafast spin dynamics and transport dedicated probing methods need to be explored. It turns out that the required spin-depth profiles, magnetic roughnesses, and magnetic height-height correlation functions are fundamentally accessible by ultrafast X-ray reflectivity and off-specular diffuse scattering experiments,^6–10^ which however are just starting to be explored.

In this paper, we report the first proof of principle ultrafast magnetic reflectivity experiment demonstrating the feasibility and the type of information that can be obtained from analyzing reflectivity results on the fs time scale.

## II. THEORY

X-rays become sensitive to element specific magnetic properties by tuning the photon energy to absorption edges as, for example, the M or L edges of the 3d transition metals. The scattering amplitude for resonant scattering in the dipolar approximation and to first order in the magnetization is^11^

$$\begin{array}{c} f=f_{0}(e_{f}^{*}\cdot e_{i})+\frac{3\lambda }{8\pi }(\left[F_{11}+F_{1-1}](e_{f}^{*}\cdot e_{i}\right)\\ -i[F_{11}-F_{1-1}](e_{f}^{*}\times e_{i})\cdot \hat{m}) \end{array},$$ (1)

where *f*_0_ is the non-magnetic Thomson scattering amplitude, *λ* is the X-ray wavelength, and $e_{i},\;e_{s}$, and $\hat{m}$ are the unit vectors representing the polarizations of the incident and scattered photons and the magnetization direction, respectively. The *F_lm_* terms are the resonant scattering amplitudes for dipolar transitions. The third term describes the effect of X-ray magnetic circular dichroism (XMCD).

Considering circularly polarized incident X-rays and denoting with *I*^+,–^ the scattered intensities observed for right (+) and left (–) circular polarization, it can be shown that the intensity difference *I*^+^ – *I*^–^ depends only on the cross-terms involving charge and magnetic scattering, i.e., on the charge-magnetic interference term. The same result occurs when reversing the direction of magnetization, given that this is possible with the sample under consideration. In reflectivity experiments, the angle of incidence *θ_i_* and detection angle *θ_f_* are the same and the scattering vector defining the length scales probed is normal to the surface with $Q_{z}=4\pi /\lambda \;\text{sin}\;(\theta )$. Denoting $A=f_{0}+3\lambda /8\pi [F_{11}+F_{1-1}]$ and $B=3\lambda /8\pi [F_{11}-F_{1-1}]$ and using the Born approximation, the difference in reflectivities for (+) and (–) circular polarization yields the charge-magnetic interference term

$$\begin{array}{c} \Delta R(q_{z})=\frac{16\pi^{2}}{q_{z}^{4}}\sum_{i=s,j=m}[\Delta g_{1,i}^{*}\Delta g_{2,j}]\text{exp}\;[-iq_{z}({\bar{z}}_{i}-{\bar{z}}_{j})]\\ \times \text{exp}\;[-\frac{1}{2}q_{z}^{2}(\sigma_{s,i}^{2}+\sigma_{m,j}^{2})]+\text{complex conjugate}, \end{array}$$ (2)

where *i *=* s* is to be summed over structural and *j *=* m* over magnetic interfaces. The scattering strength $\Delta g_{1,i}$ is the step in the quantity $N_{nr}+n_{m}A$ across the *i*th interface where *N_nr_* is the number density of non-resonant atoms times their scattering factors and *n_m_* is the number density of resonant magnetic atoms. $\Delta g_{2,j}$ is the discontinuity in the quantity $\left[n_{m}B({\hat{k}}_{f}\cdot \hat{m})+\text{cos}\;(\theta_{i}+\theta_{f})({\hat{k}}_{i}\cdot \hat{m}))\right]$ and $\sigma_{s,m}$ are the roughnesses of the structural and magnetic interfaces, respectively.

## III. EXPERIMENTAL

The expressions above point to the need for precise control of the polarization of the incoming radiation. While this is standard at synchrotron sources using variable polarization undulators, the majority of free-electron laser (FEL) sources in the soft and hard X-ray regimes operate with linearly polarized undulators for reasons such as maximizing the FEL gain or lower construction costs. The need for circularly polarized radiation is then addressed by special types of short undulators such as a DELTA-type undulator operating behind the main undulator line. FERMI is an externally seeded FEL facility based on the high-gain harmonic generation scheme (HGHG) producing intense ultrashort pulses of radiation in the VUV to XUV spectral range.^12,13^ Besides the high photon energy stability, FERMI is distinguished from the other short wavelength FELs by its use of variable-gap Apple-II undulators giving users the ability to vary and control both wavelength and polarization on times of minutes. The polarization of the VUV radiation has been measured by different methods and values varying between 0.92 and 0.97 have been reported for circularly polarized light.^14^

**FIG. 1.**
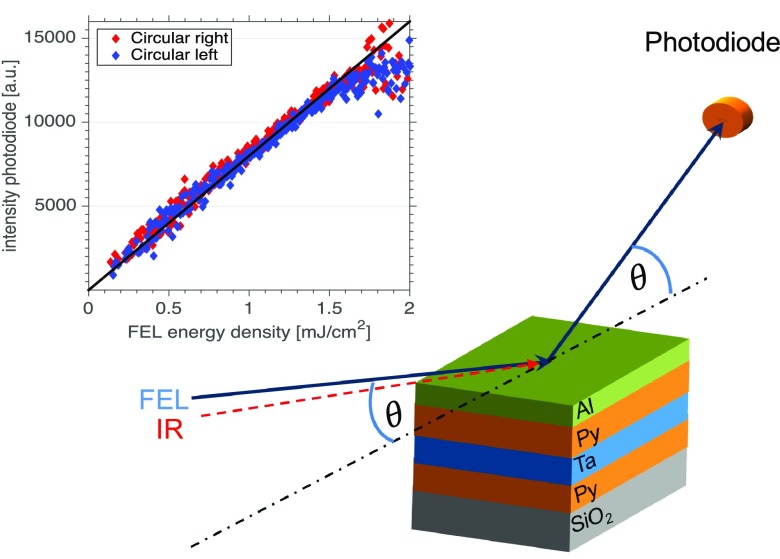
Sketch of the magnetic XUV resonant reflectivity experiment at FERMI FEL. The FEL beam and the IR beam hit the (Al/Py/Ta/Py/SiO_2_) sample in the same scattering plane with an offset of 2°. Inset: Switching of polarization: XUV intensity measured with the photodiode as a function of FEL energy density for right (red) and left (blue) circularly polarized XUV light.

The experimental setup of our XUV resonant magnetic reflectivity experiment of a magnetic multilayer excited by an ultrashort IR laser pulse is shown in Fig. 1. For the experiment, the FEL was tuned to the Fe M_2,3_-edge at a wavelength of 23.5 nm, a pulse duration of 50–60 fs, a repetition rate of 10 Hz,^15^ and a maximum pulse energy of 20 *μ*J. Using a Kirkpatrick-Baez (KB) optics, the beam was focused down to a size of 230(H) × 275(V) *μ*m^2^. Attenuators allow the adjustment of the photon flux so that the experiments can be performed below the damage threshold of the multilayer sample.^16^ The optical laser for pump-probe experiments is the same as the Ti:sapphire seed laser used for generating the FEL pulses in the HGHG scheme and therefore is intrinsically synchronized to the XUV-FEL pulses with a jitter of less than 10 fs. We used as a pump a 780 nm IR pulse of 100 fs duration and size 380(H) × 270(V) *μ*m^2^. Time delays of ± 570 ps can be achieved by a translation stage.

In reflectivity experiments, the sample is rotated with respect to a fixed incident X-ray beam. The IR and FEL beams are nearly parallel with a small angular offset of 2° which allows for a constant temporal resolution during the rotation of the sample through the beam. Moreover, such a setup avoids a second rotation stage for the IR beam. However, the parallel alignment also implies a change of IR reflectivity and penetration depth during the reflectivity scan which may need to be taken into account when modelling and interpreting the results.

Reflectivities up to scattering angles of *θ* = 60° can be measured with a reflectometer installed in the DIPROI chamber,^17,18^ corresponding to values of $Q_{z-max}$= 0.46 nm^–1^ at 23.5 nm wavelength. The reflectometer is equipped with a special Al-coated YAG/photo-diode detection system to measure the reflected XUV photons. The detector to sample distance is about 150 mm. In order to improve the sensitivity of the measuring system and avoid saturation effects due to the strong intensity of the reflected direct beam, the reflected XUV radiation is converted by a YAG scintillator screen placed in close contact to the photo-diode active surface into a longer visible light pulse via fluorescence. This light converting system is coated onto the front side of a 100 nm thick Al film which protects the photo-diode from the residual background contamination of the IR pumping laser during the time resolved part of the experiment. Since in the experimental setup the IR pumping laser impinges on the sample surface with an offset of 2° with respect to the XUV beam, an additional guard slit with an acceptance angle of 0.4° has been placed in front of the detector to minimize the optical laser background.

The spatial resolution of X-ray reflectivity experiments in the XUV regime is limited due to the rather long wavelengths. In this experimental setup, we are able to detect the charge-magnetic interference signal from interfaces that are separated by distances larger than 13.7 nm—corresponding to $d_{min}=2\pi /Q_{z,max}$. The rather high values of absorption in the XUV range are then setting a limit on the maximum distances that can be probed inside a magnetic multilayer.

The inset in Fig. 1 shows a sketch of the experimental setup and a plot of the FEL energy density versus measured photon fluxes for a series of measurements with different states of polarization [blue for (+) and red for (−) polarization]. Both helicities practically superimpose demonstrating a very reproducible switching between (+) and (−) light, which is mandatory for such experiments. FELs, even HHG seeded ones, do not provide as stable flux as synchrotrons, so measuring asymmetries with below percentage precision is a challenge and requires high statistics.

In our experimental setup, the shot to shot stability of the impinging radiation in both intensity and pointing has been monitored on-line using a four quadrant photodiode with a central clearance hole of 6 mm in diameter placed on a XY motorized stage at the entrance of the DiProI chamber (about 400 mm before the sample plane). The clearance hole is larger than the FEL beam dimension at the entrance of the experimental chamber, ensuring that only the tails of the beam profile interact with the active area of the device. This, nearly no-invasive, diagnostic acts as a local I_0_ monitor to renormalize the experimental data during the reflectivity scans and as post-processing filter to remove shots with larger intensity and pointing fluctuations when different polarizations of the FEL radiation are used. The pointing response of the four quadrant photodiode has been calibrated moving its centre of mass with respect to the incoming beam in both X and Y directions. Upon calibration, we estimate a root mean square (RMS) beam pointing instability of 1.5% in both directions of the FEL beam centre of mass, independent of the light polarization. In the post processing data analysis, only shots within one standard deviation with respect to the average FEL intensity and pointing centre of mass have been considered for the sample reflectivity curve.

The sample used in our experiment is a trilayer system with a 3 nm Al capping layer, a 12 nm thick permalloy (Py) ferromagnetic layer (Ni_81_Fe_19_), a 10 nm thick Ta spacer, and a second 12 nm thick permalloy ferromagnetic layer deposited by sputtering in a Singulus Rotaris deposition tool (Fig. 1). The second Py layer is deposited on a 100 nm thick silicon-oxide covered Si substrate. It is an in-plane magnetized system with a very low coercive field of 50 *μ*T.

## IV. RESULTS AND DISCUSSION

**FIG. 2.**
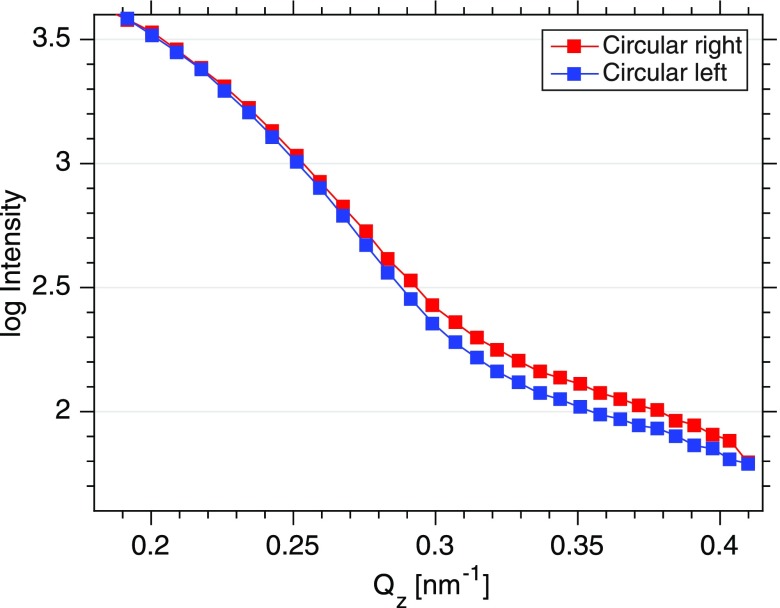
Resonant magnetic reflectivities of a permalloy (Fe_81_Ni_19_)-tantalum-permalloy trilayer system measured at the FERMI FEL. Each point is the average of 200 single shots of 50 fs pulse duration. The photon energy has been tuned to the $M_{2,3}$ edge of Fe. Reflectivities have been measured with right circularly polarized light (red) and left circularly polarized light (blue).

**FIG. 3.**
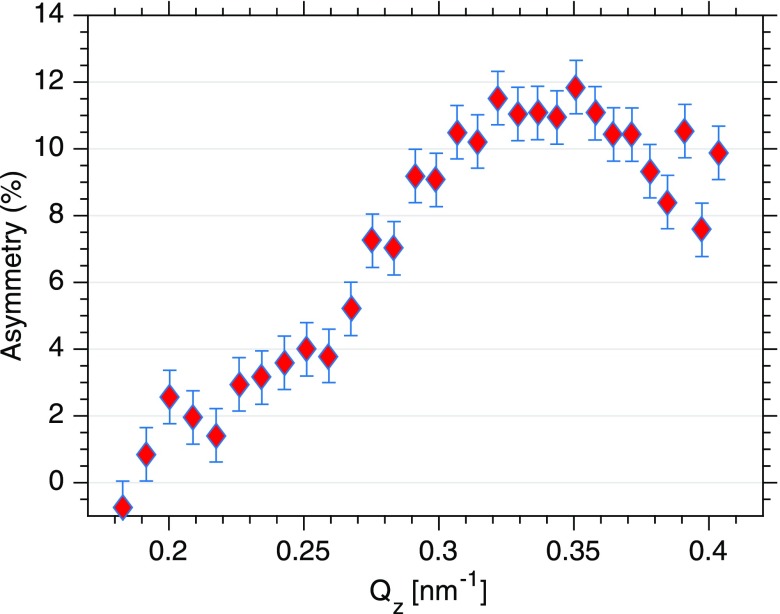
Asymmetry as deduced from the difference of the reflectivities for both photon helicities $A(Q_{z})=\Delta R(Q_{z})/(R^{+}(Q_{z})+R^{-}(Q_{z}))$.

XUV reflectivities were measured for both helicities (Fig. 2) up to a scattering angle of 50° corresponding to a wavevector transfer of *Q_z_* = 0.4 nm^–1^. The curves for left and right circularly polarized light show a pronounced Kiessig fringe resulting from the 13 nm spacing of the trilayer system. Differences between both reflectivities become apparent at *Q_z_* values exceeding 0.3 nm^–1^ evidencing the magnetic contribution to the scattering signal. The corresponding *Q*-resolved asymmetry $A(Q_{z})=\Delta R(Q_{z})/(R^{+}(Q_{z})+R^{-}(Q_{z}))$, shown in Fig. 3, is a result of the interference between the spin and charge density profiles of the trilayer system. The error for the asymmetry is determined by the number of shots *N* for each point, the average intensity $\bar{I}$, and the fluctuations of the FEL intensity expressed by its standard deviation *σ*. Consequently, the error in the resulting asymmetry can be written as $\sigma_{A}=\sigma /(\sqrt{2N}\times \bar{I})$ and is a function of the actual FEL fluctuations during the measuring interval. Thus, error bars from two similar measurements may be difficult to compare because the underlying statistics is governed not only by the average intensity but also by the actual FEL fluctuations.

The maximum value of the asymmetry is 11% ± 1%. This is consistent with the values calculated for this permalloy trilayer system with in-plane moments and optical constants known for the index of refraction of the permalloy^19^ at the Fe $M_{2,3}$ edge of $n_{\pm }=1-\delta_{0}\pm \Delta \delta +i(\beta_{0}\pm \Delta \beta )$ with $\delta_{0}=6.3\times 10^{-2},\;\Delta \delta =6.3\times 10^{-3},\;\beta_{0}=1.0\times 10^{-1}$, and $\Delta \beta =-1.6\times 10^{-2}$.

Finally, we applied ultrashort IR pulses to the magnetic trilayer system and performed a time resolved experiments. To obtain a constant pump energy density at the surface, the IR fluence was adjusted at each *Q_z_* value for the size of the footprint of the IR beam on the sample, effectively achieving an energy density of 5 mJ/cm^2^. However, the amount of absorbed IR energy is not constant when changing *Q_z_* and we calculate an increase of 40% deposited IR energy over the relevant range of *Q_z_* of the asymmetry.

**FIG. 4.**
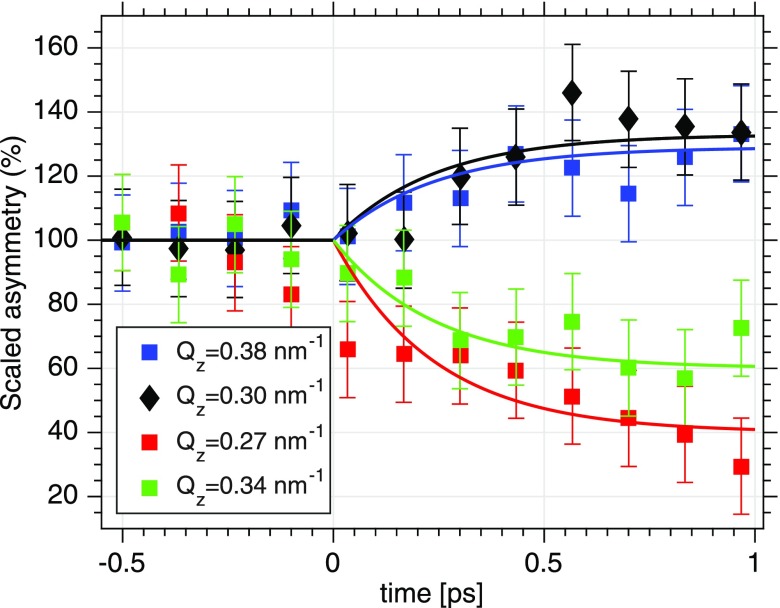
Changes to the asymmetry for four different values of *Q_z_* as a function of time delay between the IR laser and FEL. The solid line represents fits with a time constant of 240 fs for all four curves. For $Q_{z}=0.38$ nm^–1^ and *Q_z_* = 0.30 nm^–1^, we observe an ultrafast increase of the asymmetry while for *Q_z_* = 0.27 nm^–1^ and *Q_z_* = 0.34 nm^–1^ an ultrafast decrease of the asymmetry is observed.

The effect of IR pumping on the magnetic reflectivity is then demonstrated by showing pump-probe scans at selected *Q_z_* values of 0.27, 0.30, 0.34, and 0.38 nm^–^^1^ (see Fig. 4). At each *Q_z_* value, 12 time delays with a separation of 133 fs each have been measured. The onset of ultrafast IR-induced changes of the spin system becomes obvious at time delays of a few 100 fs. It is apparent that the ultrafast asymmetry changes do depend on both parameters, on the time-delay and on the *Q_z_*-values measured. The error bars here differ slightly from Fig. 4 because of the actual FEL fluctuations (see above). The black solid line represents an exponentially varying function $A(\tau )=A_{0}+\Delta A(Q_{z})(1-\text{exp}\;(-\tau /\tau_{0}))$ with the sign of the amplitude ΔA depending on *Q_z_*. The time constant *τ*_0_ is set to the previously reported demagnetization time of 240 fs for a permalloy film^20^ which fits the data. It is striking that the sign of the overall amplitude ΔA differs for different *Q_z_* values on the reflectivity curve. This implies an asymmetry that does not simply scale with the overall magnetization of the film but indicating instead the possibility of a spatially modulated spin-density profile of the multilayer upon pumping.

**FIG. 5.**
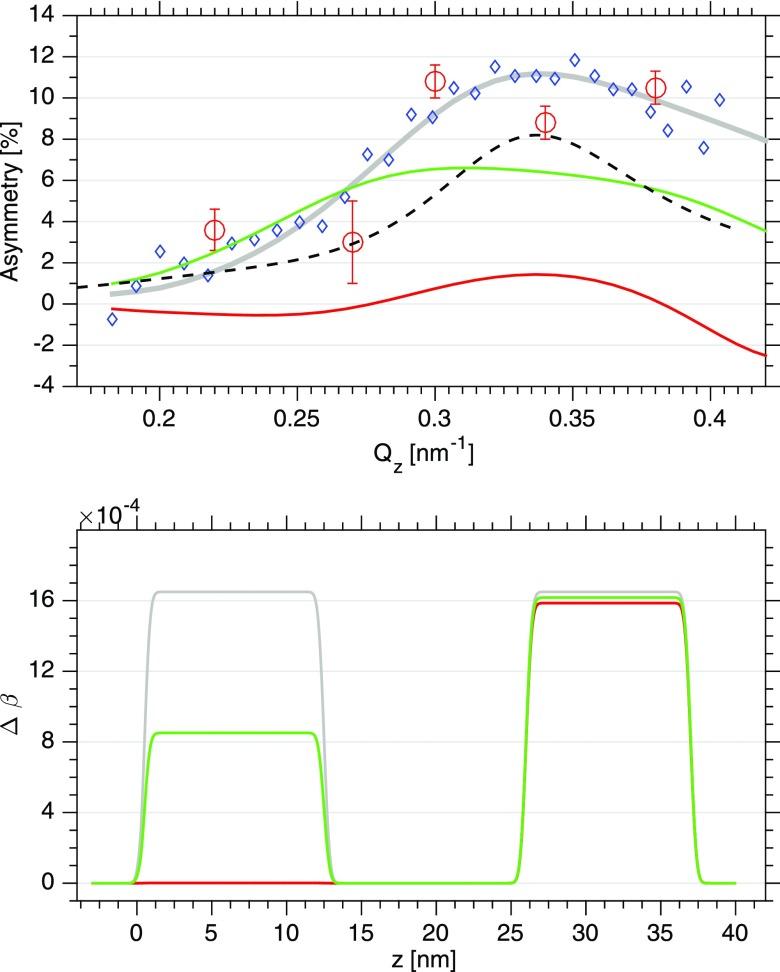
Top: (red circles) measured asymmetry values upon IR pumping at *τ *= 0.5 ps, (blue diamonds) unperturbed asymmetry from Fig. 3. The green and red lines are calculated asymmetries based on spin profiles shown on the bottom. The dashed black line displays a calculation in which a *Q_z_* dependence of the spin-profile is assumed according to the IR absorption along the reflectivity curve. Bottom: The grey line and grey spin-profile represent the spin-profile with no IR pumping. The IR beam comes from the left, the Al capping layer extends from z = −2 to 0 nm, and the Ta layer lies in between the two Py layers. The second Py layer on the right hand side is deposited on a 100 nm thick silicon-oxide covered Si substrate.

Figure 5(a) displays the unpumped asymmetry (blue circles) and all available pumped values (red circles) at a time delay of 0.5 ps. The solid lines represent calculated asymmetries for different spin profiles as shown in Fig. 5 bottom—more precisely speaking, these are profiles of the magnetic contribution Δβ to the index of refraction. The structural parameters used for modelling, such as film thickness and interface roughnesses, are based upon the results from hard and soft x-ray reflectivity experiments, respectively. Previous work showed no evidence for changes of the non-magnetic contributions *δ*_0_ and *β*_0_ to the index of refraction of Py (at the Ni-$M_{2,3}$ edge) upon IR pumping.^21^ Therefore, we restrict our modeling to adjusting the values of the dichroic absorption Δβ only. The dichroic dispersion Δδ is one order of magnitude smaller and has been found to influence the asymmetry profile not noticeable.

The undisturbed spin-profile (grey) fits the measured asymmetry quite well. The green and red spin-density profiles represent a depleted magnetization in the top-most Py layer upon pumping. The red line assumes no magnetization in the top layer and the green line a 50% reduction of the magnetization. However, in both cases the resulting asymmetries are reduced in amplitude but do not show the peculiar *Q_z_* dependence observed in the experiment. This also holds when assuming a spin depletion profile proportional to the IR power absorbed at each *Q_z_* value in the trilayer system (dashed black line). All these functions reflect a rather smooth modulation on the length scales of the Py layers which is the primary reason why they do not yield a distinct *Q_z_* modulation. In contrast, our data imply a spatial modulation of the spin-density profile with length scales inside the FM layer structure and/or a modulation of the charge interface structure upon pumping. Clearly, the small number of data points available does not allow for an extended spatial modelling of spin- and interface structure as the number of necessary fit parameters exceeds the number of data points. However, we note that spatial modulations of spin-profiles have been observed in simulations of spin-diffusion processes in magnetic multilayer systems^22,23^ and in experiments tracing spin-diffusion within magnetic domain networks.^4^ Spatial extensions reported are on the nm length scale and they persist into the ps time scale which would be consistent with our experimental data. Moreover, ultrafast changes in the charge structure as, for example, caused by coherent phonons^24^ may also alter the asymmetry curves, an effect which could be traced and separated from the spin-profile by measuring XUV reflectivities at photon energies off the resonance.

In summary, we have demonstrated that ultrafast dynamics of spin and charge density profiles in magnetic multilayer systems can be measured with ultrafast pulses from FEL sources. The full control of photon helicity allows for monitoring the corresponding *Q*-dependent asymmetry, which enables one to determine the spin-charge interference. The precision needed for the asymmetry depends on the FEL based intensity variations and on the integration time. While the limited number of data points prevents an unambiguous conclusion about the depth profile of the magnetization dynamics from our analysis here, the potential of magnetic reflectivities in determining the spatial spin distribution becomes apparent. We do not expect any fundamental obstacle for measuring high precision asymmetries at the L-edges, yielding a much higher spatial resolution.
